# Diffuse Myocardial Injuries are Present in Subclinical Hypothyroidism: A Clinical Study Using Myocardial T1-mapping Quantification

**DOI:** 10.1038/s41598-018-22970-x

**Published:** 2018-03-22

**Authors:** Zhi Yao, Xia Gao, Min Liu, Zhe Chen, Ning Yang, Yu-Mei Jia, Xiao-Meng Feng, Yuan Xu, Xin-Chun Yang, Guang Wang

**Affiliations:** 1 Department of Endocrinology, Beijing Chaoyang Hospital, Capital Medical University, Beijing, 100020 China; 2 Department of Radiology, China-Japan Friendship Hospital, Beijing, 100029 China; 3 Heart Center, Beijing Chaoyang Hospital, Capital Medical University, Beijing, 100020 China

## Abstract

Subclinical hypothyroidism (SHT) is a common disorder that may represent early thyroid dysfunction and is related to adverse cardiovascular events. However, myocardial injuries induced by SHT are difficult to detect. Our previous study demonstrated that the cardiac magnetic resonance (CMR) myocardial longitudinal relaxation time (T1) mapping technique is a useful tool for assessing diffuse myocardial injuries in overt hypothyroidism patients. This study was designed to detect whether diffuse myocardial injuries were present in SHT by using the T1 mapping technique. We found that SHT participants had significantly increased native T1 values within four segments of the left ventricle (all *p* < 0.01), especially patients with thyroid-stimulating hormone (TSH) levels ≥10 µIU/mL, compared with those in the controls. In addition, the native T1 values were negatively correlated with free thyroxine (FT4) (r = −0.476, *p* = 0.003) and were positively correlated with TSH (r = 0.489, *p* = 0.002). Furthermore, left ventricular diastolic function estimated by the peak filling rate (PFR) was significantly lower in patients with TSH levels ≥10 µIU/mL than that in the controls (*p* < 0.05). In conclusion, diffuse myocardial injuries were present in SHT, and T1 mapping may be a useful tool for evaluating mild myocardial injuries in SHT at an early stage. Our study is the first to confirm myocardial injuries in SHT patients using T1 mapping.

## Introduction

Subclinical hypothyroidism (SHT) is a very common endocrine disorder characterized by an elevated serum thyroid-stimulating hormone (TSH) concentration, but normal values of free thyroxine (FT4) and biologically active free triiodothyronine (FT3) in serum^1^. The overall prevalence of SHT in the general population varies from 4~20% and is particularly higher in women over sixty years old^2^. It is most often caused by chronic lymphocytic thyroiditis (goitrous Hashimoto’s thyroiditis and atrophic thyroiditis), an autoimmune disorder of the thyroid gland^3^, and progresses to overt hypothyroidism (HT) in approximately 5~8% of cases annually^4^. SHT is generally divided into two categories according to the TSH level: mild SHT (TSH between 4.5 and 10 µIU/mL) and severe SHT (TSH above 10 µIU/mL)^5^. Most patients with SHT have few or no typical hypothyroid symptoms. The thyroid and the cardiovascular system are closely related, both in physiological and pathological conditions^6^, and the adverse consequences of overt thyroid disease for the heart are well known. Indeed, mild forms of thyroid disease and even thyroid hormone variations within the physiological range have been linked to adverse cardiovascular effects, such as impaired diastolic relaxation and reduced exercise capacity^7^. In recent years, subclinical thyroid dysfunction has received increasing attention. The results of a 12-year study showed that heart failure events were increased in SHT patients without previous cardiovascular risks^8^. Because the potential risk factors are similar to those of HT^9,10^, some experts believe that SHT is an independent predictor for cardiovascular disease and that the risk is related to age and increased levels of TSH^11^.

In adults, thyroid hormone influences cardiac performance, including myocardial contractility, stroke volume, heart rate, vascular function and others, through genomic and non-genomic effects, thus causing changes in cardiac structure and function^12^. Therefore, when HT is present, cardiovascular function tests are typically indicated to determine whether overt cardiovascular disorders are also present, even for patients with SHT. The effects of mild thyroid hormone deficiency on the cardiovascular system have been extensively researched over the last several decades; however, most studies have focused on systolic/diastolic dysfunction. The mechanisms eliciting cardiac dysfunction in SHT are incompletely understood, but they are thought to be mediated in part by fibrosis resulting from the production of extracellular matrix proteins. Specifically, thyroid hormone deficiency can cause the accumulation of extracellular collagen and mucopolysaccharide substances in the myocardial interstitium, leading to myocardial fiber swelling, myocardial fibrosis, and myocardial oedema and then diffuse interstitial space expansion.

Given the high prevalence of SHT in the general population, and that the treatment of mild thyroid hormone deficiency is controversial^13,14^, establishing whether mild alterations of thyroid function induce myocardial injuries is important. The available evidence for the presence of myocardial tissue effects due to SHT has not been considered sufficient to warrant treatment of these subjects according to the most recent guidelines^5^. On the other hand, once a negative tissue effect is identified in an SHT patient, early aggressive treatment may have a favourable effect on cardiovascular morbidity and mortality^15^. However, the interstitial changes induced by mild thyroid hormone deficiency occur more diffusely throughout the extracellular space of the myocardium and are commonly detected by histology, which poses challenges for clinical diagnosis. Currently, a novel, validated longitudinal relaxation time (T1) mapping cardiac magnetic resonance (CMR) can be used to quantitatively assess the full range of myocardial injuries in HT that are not readily detectable by conventional CMR^16^. We previously evaluated diffuse myocardial injuries at baseline by CMR T1 mapping and found significantly increased native T1 values in overt HT patients, indicating myocardial interstitium fibrosis and oedematous lesions in the HT heart^17^. To our best knowledge, no similar studies have been conducted in the SHT population.

Therefore, this study was undertaken to detect and quantify myocardial injuries by means of T1 mapping in SHT. In addition, we investigated the relationships of both T1 values and thyroid function with LV cardiac function in these patients.

## Materials and Methods

### Patients

All research was performed in our hospital between September 2015 and March 2016. Twenty SHT patients (3 men, 17 women) with Hashimoto’s thyroiditis who were not taking levothyroxine and were between 18 and 49 years of age were invited to participate. SHT was defined as: an increased TSH level (≥4.94 µIU/mL); normal serum FT4 level; and positive anti-thyroid peroxidase (anti-TPO) antibodies. The presence of elevated anti-TPO antibodies predicts progression to overt hypothyroidism, a clear risk factor for heart disease, reflecting the importance of establishing this autoimmune phenomenon. Thyroid function should be reassessed after one month to rule out the laboratory errors or transient anomalies. Patients with known hypertension, heart disease, diabetes, liver or kidney failure, chronic pulmonary disease, tumour, adrenal insufficiency, obesity, pregnancy, connective tissue diseases, claustrophobia or metal implants were not included. Sixteen subjects (2 men, 14 women) with no documented medical history and matched for sex and age formed the healthy control group. All subjects’ vital signs and general data were recorded, including sex, age, height, body weight, blood pressure and heart rate, and their thyroid function and biochemical indexes were measured. Body mass index (BMI) and body surface area (BSA) were calculated using the following formulas: BMI (kg/m^2^) = body weight (kg)/height (m)^2^, and BSA (m^2^) = 0.007184 × height (cm)^0.725^ × weight (kg)^0.425^. Furthermore, cardiac magnetic resonance (CMR) imaging was performed. This study followed the Declaration of Helsinki guidelines and was approved by the Medical Ethics Committee of Beijing Chaoyang Hospital. All subjects provided written informed consent and ethical approval was granted for all study procedures.

### Plasma metabolites

Blood samples were taken from the peripheral vein after an overnight fast to a routine biochemical parameters analysis. FT3, FT4, TSH (Abbott Diagnostics, IL, USA), creatinine and cardiac troponin I (cTNI) levels were determined by electrochemiluminescence immunoassay (ECLIA) using an Abbott Architect i2000 (Abbott Diagnostics, Abbott Park, IL, USA). The reference intervals for FT3, FT4, TSH, cTNI and creatinine were 1.71–3.71 pg/ml, 0.7–1.48 ng/dL, 0.35–4.94 μIU/ml, 0–0.09 ng/ml and 53.0–115.0 μmol/l respectively. Total cholesterol (TC), low density lipoprotein cholesterol (LDLC), and high density lipoprotein cholesterol (HDLC) were determined using a Dade-Behring Dimension RXL Autoanalyzer (Dade Behring Diagnostics, Marburg, Germany). The reference intervals for TC, LDLC, and HDLC were 3.62–5.7 mmol/l, 1.81–3.36 mmol/l and 1.03–1.55 mmol/l respectively.

### MR protocol

All examinations were performed on a 3 T Tim Trio System scanner (Siemens Healthcare, Erlangen, Germany). CMR included cine and non-contrast T1 mapping. Cardiac volumes assessment was achieved via whole-heart coverage using gapless short-axis slices. Cardiac cines were acquired with a series of single breath-hold balanced steady-state free precession images including multiple short-axis stacks. The echo time (TE) was 1.5 ms; repetition time (TR) 3.0 ms; flip angle 50°; slice thickness was 6 mm with a 2 mm gap.

For the evaluation of diffuse myocardial injuries, Modified Look-Locker Inversion recovery (MOLLI) T1 maps^18^ were generated from the same short-axis images as previously published. Typical acquisition parameters were: TE/TR: 1.07/2.14 ms, flip angle: 35°, matrix size: 192 × 144, field of view: 340 × 255 mm, slice thickness: 6 mm, 107 phase-encoding steps, interpolated pixel size: 0.9 × 0.9, GRAPPA: 2, 24 reference lines, cardiac delay time TD: 500 ms, 206 ms acquisition time for single image, and phase partial Fourier 6/8.

### Image analysis

All MR images and maps analysis were performed offline using Argus software (Syngo MMWP workstation, Siemens AG Healthcare Sector, Forchheim, Germany) according to the Society for Cardiovascular Magnetic Resonance guidelines for reporting MR examinations^19^: LV mass (LVM), LV end-diastolic volume (EDV), LV end-systolic volume (ESV), peak ejection time (PET), peak filling time (PFT), peak ejection rate (PER), and peak filling rate (PFR). The heart rate (HR) was measured during the MRI procedure. All volumetric indexes and function parameters were normalized to the BSA. The LVM index (g/m^2^) was calculated by multiplying the difference between the epicardial and endocardial end-diastolic indexed volumes by the density of the myocardial tissue (1.05 g/mL). Analyses of the T1 relaxation maps were performed directly on the maps as described in a previous study^20^. Short-axis images were automatically contoured to outline the endocardium and epicardium. To quantify the T1 values, manual contouring was used to define the regions of interest (ROIs) in the anterior, septal, inferior and lateral segments of the LV myocardium on the mid-ventricular short- axis slices. Shimming and center frequency adjustments were performed to generate images free from off-resonance artifacts. All quantitative analyses of all CMR images were performed by two operators unaware of the subjects’ clinical information and the results of other diagnostic tests.

### Statistical analysis

Continuous data were tested for normality using the Kolmogorov-Smirnov test. Normal distribution data were expressed as means ± standard deviation (SD) and abnormal distribution data were expressed as medians (25th and 75th percentiles). The comparison between the two groups utilized unpaired Student t-test, or Mann-Whitney U test (nonparametric test). Multiple comparisons were performed by One-way ANOVA, with post hoc testing as appropriate. Correlations of variables were determined by Spearman or Pearson’s analysis. All of the statistical tests were two-tailed and *p* < 0.05 was considered statistically significant. All data analyses were conducted using SPSS Statistics (version 21.0, Chicago, IL).

## Results

### Clinical and hormonal characteristics

A total of 36 subjects were evaluated during the study period, including 20 patients with SHT and 16 controls. The baseline characteristics of the two groups are reported in Table 1. Of the SHT patients, 12.5% were male compared to 15% of the control subjects. The SHT patients and the controls had similar ages. No significant differences were observed in blood pressure (BP), BMI, heart rate (HR), TC, LDLC, HDLC, cTNI and creatinine levels between the SHT and control groups (all *p* > 0.05). FT3 levels in the SHT patients were slightly lower compared with those in the controls (2.61 ± 0.38 vs. 2.83 ± 0.43 pg/ml, *p* = 0.125), but the difference did not reach statistical significance. The TSH level was significantly higher in the SHT group comparison with that in the control group [8.99 (6.54–22.99) vs. 1.78(1.41–3.22) µIU/ml, *p* < 0.0001]. Notably, although they remained within the normal range, the FT4 levels of the SHT patients were significantly lower than those in the controls (0.89 ± 0.19 vs. 1.10 ± 0.14 ng/dl, *p* = 0.001), indicating thyroid hormone deficiency.

**Table 1:** Baseline Characteristics of Control and SHT groups.

	Controls (n = 16)	SHT (n = 20)	p value
Males, n (%)	2 (12.5%)	3 (15%)	1.00
Age, years	35.56 ± 8.78	36.85 ± 8.46	0.658
SBP, mmHg	115.31 ± 9.77	118.20 ± 7.84	0.784
DBP, mmHg	67.50 ± 5.48	72.25 ± 5.95	0.991
BMI, kg/m^2^	22.36 ± 2.89	23.48 ± 4.48	0.393
HR, beats/min	67.75 ± 9.18	69.11 ± 10.21	0.685
TC, mmol/l	4.51 ± 0.80	4.93 ± 1.28	0.284
LDLC, mmol/l	2.52 ± 0.85	2.82 ± 0.96	0.358
HDLC, mmol/l	1.72 ± 0.38	1.57 ± 0.34	0.257
FT3, pg/ml	2.83 ± 0.43	2.61 ± 0.38	0.125
FT4, ng/dl	1.10 ± 0.14	0.89 ± 0.19	*0.001*
TSH, µIU/ml	1.78 (1.41–3.22)	8.99 (6.54–22.99)	*<0.0001*
cTNI, ng/dl	0 (0–0)	0 (0–0.025)	0.666
Creatinine, µmol/l	61.83 ± 15.07	70.41 ± 15.40	0.812

Abbreviations: SBP, systolic blood pressure; DBP, diastolic blood pressure; BMI, body mass index; HR, heart rate; TC, total cholesterol; LDLC, low density lipoprotein cholesterol; HDLC, high density lipoprotein cholesterol; FT3, free triiodothyronine; FT4, free thyroxine; TSH, thyroid stimulating hormone; cTNI, cardiac troponin I.

### Cardiac magnetic resonance findings

CMR was successfully completed in all 36 subjects and the results are displayed in Table 2. No statistically significant differences were found in BSA, volumetric indexes (EDV, ESV and LVMI), or any parameters of systolic and diastolic function (EF, PET, PFT, SV, CI, PER and PFR) between the control and SHT groups. Considering that the minimal alterations of thyroid status observed in the SHT patients with TSH levels <10 µIU/ml (mild SHT) were not sufficient to lead to any marked changes in LV morphology and function, we further compared the controls and a subgroup of SHT patients and found that the PFR was significantly decreased in patients with TSH levels ≥10 µIU/ml (severe SHT) compared to that in the controls (3.22 ± 0.79 vs. 4.24 ± 1.08 EDV/s, *p* = 0.026), suggesting poor cardiac filling in individuals with early thyroid hormone deficiency. (Table 3).

**Table 2:** Cardiovascular Magnetic Resonance Parameters of Control and SHT groups.

	Controls (n = 16)	SHT (n = 20)	p value
BSA, m^2^	1.67 ± 0.18	1.67 ± 0.17	0.897
EF, %	61.14 ± 5.05	63.61 ± 6.02	0.203
PET, ms	138.73 ± 25.91	120.14 ± 34.66	0.086
PFT, ms	137.41 ± 23.53	133.47 ± 37.21	0.723
EDV, ml/m^2^	57.53 ± 11.68	53.91 ± 10.85	0.349
ESV, ml/m^2^	21.94 ± 6.76	20.46 ± 6.12	0.503
SV, ml/m^2^	35.66 ± 6.62	33.35 ± 6.46	0.305
CI, l/min/m^2^	2.38 ± 0.37	2.29 ± 0.44	0.492
LVMI, g/ m^2^	50.91 ± 11.61	51.78 ± 11.32	0.825
PER, EDV/S	3.48 ± 0.46	3.61 ± 0.56	0.450
PFR, EDV/s	4.24 ± 1.08	3.89 ± 1.05	0.343
T1-LVAW, ms	1031.20 ± 53.17	1097.07 ± 69.37	*0.004*
T1-IVS, ms	1074.90 ± 34.20	1139.80 ± 79.04	*0.004*
T1-LVIW, ms	1056.14 ± 44.03	1117.04 ± 76.73	*0.008*
T1-LVLW, ms	1058.69 ± 45.30	1129.04 ± 80.20	*0.004*

Abbreviations: BSA, body surface area; EF, ejection fraction; PET, peak ejection time; PFT, peak filling time; EDV, end diastolic volume; ESV, end systolic volume; SV, stroke volume; CI, cardiac index; LVMI, left ventricular mass index; PER, peak ejection rate; PFR, peak filling rate; LVAW, left-ventricular anterior wall; IVS, interventricular septum; LVIW, left-ventricular inferior wall; LVLW, left-ventricular lateral wall.

**Table 3:** Cardiovascular Magnetic Resonance Parameters of Control and subgroup of SHT.

	Controls (n = 16)	TSH ≥ 10 (n = 10)	p value
EF, %	61.14 ± 5.05	63.24 ± 7.95	0.426
PET, ms	138.73 ± 25.91	121.40 ± 33.77	0.164
PFT, ms	137.41 ± 23.53	137.40 ± 44.73	0.999
EDV, ml/m^2^	57.53 ± 11.68	51.70 ± 10.83	0.232
ESV, ml/m^2^	21.94 ± 6.76	20.18 ± 5.58	0.514
SV, ml/m^2^	35.66 ± 6.62	31.41 ± 6.77	0.141
CI, l/min/m^2^	2.38 ± 0.37	2.22 ± 0.55	0.375
LVMI, g/ m^2^	50.91 ± 11.61	49.50 ± 13.15	0.783
PER, EDV/S	3.48 ± 0.46	3.58 ± 0.45	0.569
PFR, EDV/s	4.24 ± 1.08	3.22 ± 0.79	*0.026*

Abbreviations: EF, ejection fraction; PET, peak ejection time; PFT, peak filling time; EDV, end diastolic volume; ESV, end systolic volume; SV, stroke volume; CI, cardiac index; LVMI, left ventricular mass index; PER, peak ejection rate; PFR, peak filling rate.

Based on four segments (anterior, septal, inferior and lateral) in the LV, native MOLLI showed significantly higher myocardial T1 values in the SHT patients compared with those in the healthy controls (all *p* < 0.01) (Table 2; Table 5). Subgroup analysis showed that T1 values within the LV inferior wall (LVIW) and lateral wall (LVLW) were even higher in the severe SHT patients compared with those in the mild SHT individuals (1150.45 ± 73.62 vs. 1083.62 ± 67.28 ms and 1160.82 ± 88.13 vs. 1097.25 ± 59.72 ms separately; all *p* < 0.05). However, no significant differences in T1 values were observed between the mild SHT subgroup and the control group (all *p* > 0.05) (Table 4).

**Table 4:** T1 values of Control group and Two Subgroups in SHT.

	Controls	Subclinical Hypothyroid group (n = 20)	
	(n = 16)	TSH < 10 (n = 10)	TSH ≥ 10 (n = 10)
T1-LVAW, ms	1031.20 ± 53.17	1079.05 ± 64.60	1115.09 ± 72.57^**^
T1-IVS, ms	1074.90 ± 34.20	1117.28 ± 80.04	1162.31 ± 75.21^**^
T1-LVIW, ms	1056.14 ± 44.03	1083.62 ± 67.28	1150.45 ± 73.62^**,#^
T1-LVLW, ms	1058.69 ± 45.30	1097.25 ± 59.72	1160.82 ± 88.13^**,#^

Abbreviations: LVAW, left-ventricular anterior wall; IVS, interventricular septum; LVIW, left-ventricular inferior wall; LVLW, left-ventricular lateral wall. **P < 0.01 vs. the control group; ^#^P < 0.05 vs. TSH < 10 µIU/ml subgroup.

**Table 5:** Frequency Distribution of T1 Value in control group and two subgroups of SHT.

T1 value	LVAW			IVS			LVIW			LVLW		
(msec)	Con	TSH < 10	TSH > 10	Con	TSH < 10	TSH > 10	Con	TSH < 10	TSH > 10	Con	TSH < 10	TSH > 10
900–1000	3							1				
1000–1100	11	6	4	15	5	1	15	3	1	13	6	3
1100–1200	2	4	5	1	3	6	1	6	8	3	3	4
1200–1300			1		2	3			1		1	3

LVAW, left-ventricular anterior wall; IVS, interventricular septum; LVIW, left-ventricular inferior wall; LVLW, left-ventricular lateral wall.

### Native myocardial T1 imaging

**Figure 1:**
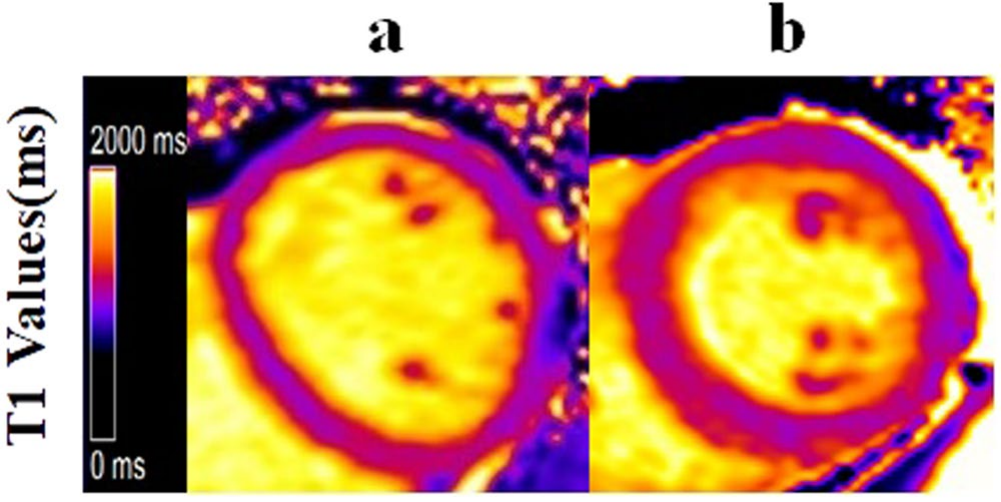
Color maps of the T1 values based on a modified Look-Locker inversion in a mid-ventricular short-axis slice acquired at 3.0 T. (**a**) Normal control, (**b**) SHT. Note the marked elevation in the myocardial T1 value in the patient with SHT(b) (T1 = 1202 msec) compared with the normal control (**a**) (T1 = 1030 msec).

Native MOLLI (Fig. 1) showed significantly higher native myocardial T1 values in a SHT patient compared with a healthy control.

### Correlation between myocardial native T1 values and thyroid function

**Figure 2:**
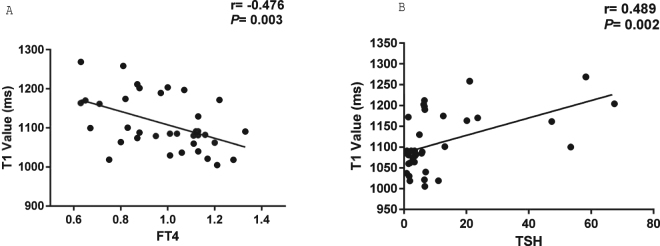
Bivariate analysis of the correlation between T1 values of IVS and thyroid function in control group and SHT group. Spearman or Pearson analysis was used to assess the correlation between T1 values and FT4 (**A**) and TSH (**B**). The central line represents the regression line.

Due to showing the least segmental variation, the T1 value of the septal segment was used as an indicator of myocardial injury to investigate the relationships between T1 values and thyroid function. A negative correlation was observed between myocardial T1 time and FT4 (Pearson correlation, r = −0.476, *p* = 0.003) (Fig. 2A), and a significant positive correlation was found between T1 values and TSH (Spearman correlation, r = −0.489, *p* = 0.002) (Fig. 2B). These findings suggest that thyroid hormone deficiency, even at an early stage, is associated with myocardial injury. In addition, no correlation was found between T1 time and cardiac function.

## Discussion

This was the first study in which the index of myocardial injury (fibrosis and oedema) was evaluated in patients with subclinical hypothyroidism. There are several important findings in our study: SHT patients have significantly longer native myocardial T1 times compared to those of healthy controls, which is consistent with the presence of diffuse interstitial myocardial fibrosis. Furthermore, the extent of this fibrosis was correlated with the levels of FT4 and TSH, suggesting a hormone link between diffuse myocardial injuries and abnormal thyroid function. In addition, the patients with severe SHT were affected by diastolic dysfunction as evidenced by a significantly reduced PFR compared to that in the controls. The patients with TSH levels between 4.5 and 10 µIU/ml were not affected by myocardial injury.

The effect of altered thyroid function is apparent in clinical practice by the cardiovascular alterations observed in patients with SHT^21^. Previous ultrasonic studies have shown that the mitral diastolic peak late velocity (Amax), mitral diastolic early inflow deceleration time and isometric relaxation time (IRT) were significantly higher in SHT patients versus those in controls^22^, all of which are parameters of left ventricular diastolic function. Importantly, impaired diastolic function often precedes systolic dysfunction in HT patients. Additionally, SHT is regarded as an independent risk factor of coronary heart disease (CHD)^23,24^.

CMR is currently the most accurate and reproducible technique for evaluating cardiac volumes and function due to its excellent image resolution and intrinsic tissue contrast. Previous research utilizing CMR reported that only cardiac dysfunction, such as typical diastolic dysfunction, was induced by thyroid hormone deficiency in patients with overt HT^25^. Despite thyroid hormones within the normal range, SHT should be considered a mild form of thyroid failure, in which impaired diastolic relaxation has also been documented^26^. However, no evidence is available to support the presence of tissue effects due to altered thyroid function in SHT patients. T1 mapping is a previously histologically validated CMR technique that can provide information regarding myocardial injury in different ventricular regional segments^27^. CMR with T1 mapping quantification allows detection of coexisting functional and textual abnormalities of the myocardium that are partially undetectable by conventional CMR in patients with mild thyroid hormone deficiency. Our research demonstrated by quantification that diffuse myocardial injuries could be detected in SHT even in the absence of clinical symptoms for the first time. Meanwhile, myocardial abnormalities were associated with disease severity in patients with SHT.

The cellular mechanisms by which thyroid hormones act on diastolic cardiac function are complex. In animal experiments, SHT in adult hamsters resulted in thinning of myocardial artery, decrease myocardial cells and extensive fibrosis^28^. Thyroid hormones modulate the expression and function of several enzymes and proteins involved in cardiac performance. Thyroid hormone deficiency can result in reduced calcium reuptake into the sarcoplasmic reticulum during diastole and increased hyaluronan in the myocardial interstitium, which can impair diastolic filling. An age-related decline in LV diastolic function should also be considered. However, no difference in age was found between the SHT patients and the controls in our study.

Further analysis confirmed the negative relationship between thyroid hormone deficiency and myocardial injuries. The serum TSH level changes logarithmically with arithmetic changes in the serum concentration of FT4^29^. Therefore, a mild decrease in the serum FT4 concentration within the normal range could cause the TSH level to increase beyond its normal range. Most experts believe that an elevated serum TSH concentration is a marker of true thyroid hormone deficiency. Dr. Sellitti detected clear TSH receptor (TSHR) mRNA expression in a pig’s heart by RT-PCR technology. Dr. ZhaoJiajun reported that the heart was the target organ of TSH because he identified the presence of functional TSHRs in the ventricular tissue cells of mice. However, the mechanism of the characteristically elevated serum TSH level in SHT in the induction of cardiac damage is not yet clear.

In previous studies, impaired LV diastolic function is the most consistent finding in patients with SHT as confirmed by Doppler echocardiography, TDE, CMR and radionuclide ventriculography (RNV)^30^. The relative levels of fibrosis varied markedly between the severe SHT patients and the controls in our study, which may partially account for the decreased PFR, thus explaining the impaired ventricular filling at an early stage. Moreover, MRI-derived indexes of LV diastolic filling revealed abnormalities of myocardial relaxation only in patients with severe SHT and not in those with mild SHT in our research. The cardiac effects of subtle thyroid dysfunction, such as mild SHT, are less clear. In a recent study, CMR was used to evaluate 30 women with mild SHT (TSH: 3.7–8.7 µIU/ml) due to Hashimoto’s thyroiditis and 20 matched control subjects. The EDV was significantly decreased and systolic performance was altered in these mild SHT patients^31^. Furthermore, no significant differences were observed, but significant overlap of native T1 times was identified between the mild SHT patients and the control subjects, implying a limited extent of diffuse myocardial fibrosis in mild SHT. The relationship between native myocardial T1 times and diastolic dysfunction has been described in overt HT patients in our previous study^17^. However, we did not observe a significant correlation between T1 values and PFR in this study, which is regarded as a reflection of LV diastolic filling. This result may be due to differences in the numbers and conditions of the patients enrolled.

Currently, oral L-thyroxine replacement therapy for SHT patients is controversial because the benefits of this therapy vary across different situations^32,33^. Whether levothyroxine could be cardiotoxic is also controversial. Previous animal experiments have shown that in adult hamsters with SHT, thyroid hormone replacement therapy could prevent myocyte loss, reverse left ventricular remodeling, and improve coronary flow^34^. Furthermore, in a rat myocardial infarction-heart model, Martin Gerdes’ group found that thyroid hormone replacement therapy attenuated atrial remodeling and reduced atrial fibrillation inducibility^35^. In clinical practice, early detection of myocardial lesions is critical to the timing of treatment. Based on the natural course of the disease, oral L-thyroxine replacement therapy is recommended for SHT patients with TSH levels >10 µIU/ml^29^. For SHT patients with TSH levels <10 µIU/ml, the American Association of Clinical Endocrinologists (AACE) and the American College of Endocrinology (ACE) both agree that the decision to treat should be tailored to each individual patient without any standardization. A study from the Cleveland Clinic Preventative Cardiology Clinic showed that untreated patients with TSH levels greater than 6.1 µIU/ml had higher all-cause mortality^36^. Another study on adults with atrial fibrillation (AF) with or without levothyroxine treatment showed that levothyroxine treatment decreased the mortality risk in women with AF and reported that the treatment seemed to be safe^37^. According to our study, we found that severe SHT was associated with obvious myocardial injuries by T1 mapping CMR and should be treated. This conclusion is consistent with clinical treatment guidelines. For mild SHT, if T1 values increase obviously, we suggest that timely treatment should be applied. Therefore, the ability to non-invasively evaluate diffuse fibrosis in SHT is likely to enhance our understanding of the pathogenesis and disease progression. Additionally, T1 quantification can be used for early detection and can serve as a screening tool before overt myocardial changes manifest. The age of SHT patients should also be mentioned. With increasing age, the range of normal TSH levels may be wider. Therefore, elderly individuals with mild TSH elevations may not actually qualify as subjects with subclinical hypothyroidism. The patients in our study were young and may benefit from CMR to detect SHT-induced cardiac dysfunction.

### Study limitations

Our study was a single-center study with a relatively small number of enrolled patients. Furthermore, since the time at which the active pro-fibrotic state manifests in the myocardium of patients with SHT is uncertain, serial imaging using T1 mapping techniques over a patient’s lifetime may provide crucial information regarding the timing of this process. However, our conclusions require a study with a larger sample size for further confirmation.

## Conclusions

SHT has an insidious onset without typical symptoms and increases the incidence of adverse cardiovascular events. Several important cardiovascular risk factors may improve with L-T4 replacement therapy^38^. Therefore, early detection of myocardial injuries caused by SHT is particularly important. Our study confirmed the presence of myocardial injuries in patients with SHT using T1 mapping for the first time, especially in those with severe SHT. The data summarized in this paper strongly support treatment of these patients. As an emerging tool, CMR T1 mapping can help us detect subclinical myocardial lesions earlier and provide a basis for individual treatment plans. We believe that this technique has good clinical application value and may be more common in the near future.
